# 217. Antimicrobial Activity of Dalbavancin against Gram-Positive Bacteria Isolated from Patients Hospitalized with Bacteremia in United States and European Medical Centers: Results from the International Dalbavancin Evaluation of Activity (IDEA) Program (2018-202)

**DOI:** 10.1093/ofid/ofab466.419

**Published:** 2021-12-04

**Authors:** Helio S Sader, Mariana Castanheira, Mariana Castanheira, Michael D Huband, Dee Shortridge, Cecilia G Carvalhaes, Rodrigo E Mendes

**Affiliations:** 1 JMI Laboratories, North Liberty, Iowa; 2 JMI Labortories, North Liberty, Iowa; 3 JMI Laboratories, Inc., North Liberty, Iowa

## Abstract

**Background:**

The IDEA Program monitors the *in vitro* activity of dalbavancin and comparators against Gram-positive (GP) bacteria causing bloodstream infections (BSIs) and other infections in the United States (US) and Europe (EU). We evaluated the BSI results in 2018-2020.

**Methods:**

8,643 organisms were consecutively collected (1/patient) from 74 medical centers located in the US (n=4,544; 33 centers), western EU (W-EU; n=3,330; 28 centers from 10 nations: Belgium, France, Germany, Ireland, Italy, Portugal, Spain, Sweden, Switzerland, and the United Kingdom), and eastern EU (E-EU; n=769; 13 centers from 10 nations: Belarus, Czech Republic, Greece, Hungary, Israel, Poland, Romania, Russia, Slovenia, and Turkey). Organisms were susceptibility tested by reference broth microdilution methods in a central laboratory.

**Results:**

Overall, the most common GP organisms were *S. aureus* (SA; 45.2%), *E. faecalis* (EF; 12.2%), *S. epidermidis* (SEP; 8.9%), β-hemolytic streptococci (BHS; 8.5%), and *E. faecium* (EFM; 7.6%), but rank order varied markedly by geographic region. The top 3 GP organisms were SA, EF, and BHS in the US; SA, EF, and EFM in W-EU; and SA, *S. pneumoniae*, and BHS in E-EU. Dalbavancin was highly active against methicillin-susceptible (MSSA) and -resistant (MRSA) SA, with an MIC_90_ of 0.03 mg/L in all 3 regions (Table). Among SA, MRSA rates were higher in the US (41.3%) than W-EU (21.5%) or E-EU (27.3%), and ceftaroline susceptibility ranged from 95.4% (W-EU) to 96.6% (US). Vancomycin (VAN) susceptibility varied from 97.3% (E-EU) to 98.3% (W-EU) among EF (97.5% in US), and dalbavancin was active against all VAN-S EF (MIC_50/90_, 0.03/0.06 mg/L; 100.0%S). Among SEP, all isolates were inhibited at ≤0.25 mg/L of dalbavancin (MIC_50/90_, 0.03/0.06 mg/L) and oxacillin resistance ranged from 66.9% in W-EU to 86.5% in E-EU (73.2% in US). BHS exhibited low dalbavancin MIC values (MIC_50/90_, 0.015/0.03 mg/L) and high S rates for its comparators. Among EFM, VAN-S rates varied from 36.6% in the US to 61.6% in E-EU and 76.1% in W-EU, and dalbavancin inhibited all VAN-S EFM at ≤0.25 mg/L (MIC_50/90_, 0.03/0.12 mg/L).

**Conclusion:**

Dalbavancin exhibited potent activity and broad spectrum against a large collection of contemporary GP bacteria recovered from patients with BSI in US and EU.

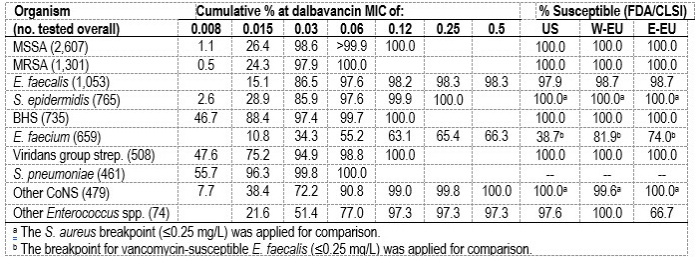

**Disclosures:**

**Helio S. Sader, MD, PhD, FIDSA**, **AbbVie (formerly Allergan**) (Research Grant or Support)**Basilea Pharmaceutica International, Ltd.** (Research Grant or Support)**Cipla Therapeutics** (Research Grant or Support)**Cipla USA Inc.** (Research Grant or Support)**Department of Health and Human Services** (Research Grant or Support, Contract no. HHSO100201600002C)**Melinta Therapeutics, LLC** (Research Grant or Support)**Nabriva Therapeutics** (Research Grant or Support)**Pfizer, Inc.** (Research Grant or Support)**Shionogi** (Research Grant or Support)**Spero Therapeutics** (Research Grant or Support) **Mariana Castanheira, PhD**, **AbbVie (formerly Allergan**) (Research Grant or Support)**Bravos Biosciences** (Research Grant or Support)**Cidara Therapeutics, Inc.** (Research Grant or Support)**Cipla Therapeutics** (Research Grant or Support)**Cipla USA Inc.** (Research Grant or Support)**GlaxoSmithKline** (Research Grant or Support)**Melinta Therapeutics, Inc.** (Research Grant or Support)**Melinta Therapeutics, LLC** (Research Grant or Support)**Pfizer, Inc.** (Research Grant or Support)**Qpex Biopharma** (Research Grant or Support)**Shionogi** (Research Grant or Support)**Spero Therapeutics** (Research Grant or Support) **Mariana Castanheira, PhD**, Affinity Biosensors (Individual(s) Involved: Self): Research Grant or Support; Allergan (Individual(s) Involved: Self): Research Grant or Support; Amicrobe, Inc (Individual(s) Involved: Self): Research Grant or Support; Amplyx Pharma (Individual(s) Involved: Self): Research Grant or Support; Artugen Therapeutics USA, Inc. (Individual(s) Involved: Self): Research Grant or Support; Astellas (Individual(s) Involved: Self): Research Grant or Support; Basilea (Individual(s) Involved: Self): Research Grant or Support; Beth Israel Deaconess Medical Center (Individual(s) Involved: Self): Research Grant or Support; BIDMC (Individual(s) Involved: Self): Research Grant or Support; bioMerieux Inc. (Individual(s) Involved: Self): Research Grant or Support; BioVersys Ag (Individual(s) Involved: Self): Research Grant or Support; Bugworks (Individual(s) Involved: Self): Research Grant or Support; Cidara (Individual(s) Involved: Self): Research Grant or Support; Cipla (Individual(s) Involved: Self): Research Grant or Support; Contrafect (Individual(s) Involved: Self): Research Grant or Support; Cormedix (Individual(s) Involved: Self): Research Grant or Support; Crestone, Inc. (Individual(s) Involved: Self): Research Grant or Support; Curza (Individual(s) Involved: Self): Research Grant or Support; CXC7 (Individual(s) Involved: Self): Research Grant or Support; Entasis (Individual(s) Involved: Self): Research Grant or Support; Fedora Pharmaceutical (Individual(s) Involved: Self): Research Grant or Support; Fimbrion Therapeutics (Individual(s) Involved: Self): Research Grant or Support; Fox Chase (Individual(s) Involved: Self): Research Grant or Support; GlaxoSmithKline (Individual(s) Involved: Self): Research Grant or Support; Guardian Therapeutics (Individual(s) Involved: Self): Research Grant or Support; Hardy Diagnostics (Individual(s) Involved: Self): Research Grant or Support; IHMA (Individual(s) Involved: Self): Research Grant or Support; Janssen Research & Development (Individual(s) Involved: Self): Research Grant or Support; Johnson & Johnson (Individual(s) Involved: Self): Research Grant or Support; Kaleido Biosceinces (Individual(s) Involved: Self): Research Grant or Support; KBP Biosciences (Individual(s) Involved: Self): Research Grant or Support; Luminex (Individual(s) Involved: Self): Research Grant or Support; Matrivax (Individual(s) Involved: Self): Research Grant or Support; Mayo Clinic (Individual(s) Involved: Self): Research Grant or Support; Medpace (Individual(s) Involved: Self): Research Grant or Support; Meiji Seika Pharma Co., Ltd. (Individual(s) Involved: Self): Research Grant or Support; Melinta (Individual(s) Involved: Self): Research Grant or Support; Menarini (Individual(s) Involved: Self): Research Grant or Support; Merck (Individual(s) Involved: Self): Research Grant or Support; Meridian Bioscience Inc. (Individual(s) Involved: Self): Research Grant or Support; Micromyx (Individual(s) Involved: Self): Research Grant or Support; MicuRx (Individual(s) Involved: Self): Research Grant or Support; N8 Medical (Individual(s) Involved: Self): Research Grant or Support; Nabriva (Individual(s) Involved: Self): Research Grant or Support; National Institutes of Health (Individual(s) Involved: Self): Research Grant or Support; National University of Singapore (Individual(s) Involved: Self): Research Grant or Support; North Bristol NHS Trust (Individual(s) Involved: Self): Research Grant or Support; Novome Biotechnologies (Individual(s) Involved: Self): Research Grant or Support; Paratek (Individual(s) Involved: Self): Research Grant or Support; Pfizer (Individual(s) Involved: Self): Research Grant or Support; Prokaryotics Inc. (Individual(s) Involved: Self): Research Grant or Support; QPEX Biopharma (Individual(s) Involved: Self): Research Grant or Support; Rhode Island Hospital (Individual(s) Involved: Self): Research Grant or Support; RIHML (Individual(s) Involved: Self): Research Grant or Support; Roche (Individual(s) Involved: Self): Research Grant or Support; Roivant (Individual(s) Involved: Self): Research Grant or Support; Salvat (Individual(s) Involved: Self): Research Grant or Support; Scynexis (Individual(s) Involved: Self): Research Grant or Support; SeLux Diagnostics (Individual(s) Involved: Self): Research Grant or Support; Shionogi (Individual(s) Involved: Self): Research Grant or Support; Specific Diagnostics (Individual(s) Involved: Self): Research Grant or Support; Spero (Individual(s) Involved: Self): Research Grant or Support; SuperTrans Medical LT (Individual(s) Involved: Self): Research Grant or Support; T2 Biosystems (Individual(s) Involved: Self): Research Grant or Support; The University of Queensland (Individual(s) Involved: Self): Research Grant or Support; Thermo Fisher Scientific (Individual(s) Involved: Self): Research Grant or Support; Tufts Medical Center (Individual(s) Involved: Self): Research Grant or Support; Universite de Sherbrooke (Individual(s) Involved: Self): Research Grant or Support; University of Iowa (Individual(s) Involved: Self): Research Grant or Support; University of Iowa Hospitals and Clinics (Individual(s) Involved: Self): Research Grant or Support; University of Wisconsin (Individual(s) Involved: Self): Research Grant or Support; UNT System College of Pharmacy (Individual(s) Involved: Self): Research Grant or Support; URMC (Individual(s) Involved: Self): Research Grant or Support; UT Southwestern (Individual(s) Involved: Self): Research Grant or Support; VenatoRx (Individual(s) Involved: Self): Research Grant or Support; Viosera Therapeutics (Individual(s) Involved: Self): Research Grant or Support; Wayne State University (Individual(s) Involved: Self): Research Grant or Support **Michael D. Huband, BS**, **AbbVie (formerly Allergan**) (Research Grant or Support)**Melinta Therapeutics, LLC** (Research Grant or Support) **Dee Shortridge, PhD**, **AbbVie (formerly Allergan**) (Research Grant or Support)**Melinta Therapeutics, Inc.** (Research Grant or Support)**Melinta Therapeutics, LLC** (Research Grant or Support)**Shionogi** (Research Grant or Support) **Cecilia G. Carvalhaes, MD, PhD**, **AbbVie (formerly Allergan**) (Research Grant or Support)**Cidara Therapeutics, Inc.** (Research Grant or Support)**Cipla Therapeutics** (Research Grant or Support)**Cipla USA Inc.** (Research Grant or Support)**Melinta Therapeutics, LLC** (Research Grant or Support)**Pfizer, Inc.** (Research Grant or Support) **Rodrigo E. Mendes, PhD**, **AbbVie** (Research Grant or Support)**AbbVie (formerly Allergan**) (Research Grant or Support)**Cipla Therapeutics** (Research Grant or Support)**Cipla USA Inc.** (Research Grant or Support)**ContraFect Corporation** (Research Grant or Support)**GlaxoSmithKline, LLC** (Research Grant or Support)**Melinta Therapeutics, Inc.** (Research Grant or Support)**Melinta Therapeutics, LLC** (Research Grant or Support)**Nabriva Therapeutics** (Research Grant or Support)**Pfizer, Inc.** (Research Grant or Support)**Shionogi** (Research Grant or Support)**Spero Therapeutics** (Research Grant or Support)

